# Nickel excess affects phenology and reproductive attributes of *Asterella wallichiana* and *Plagiochasma appendiculatum* growing in natural habitats

**DOI:** 10.1038/s41598-020-73441-1

**Published:** 2021-02-09

**Authors:** Anil Sharma, Madhu Bhagat, Mohammad Urfan, Bilal Ahmed, Anima Langer, Villayat Ali, Dhiraj Vyas, Narendra Singh Yadav, Haroon Rashid Hakla, Shubham Sharma, Sikander Pal

**Affiliations:** 1 Department of Botany, University of Jammu, Jammu, 180-006 India; 2 Biodiversity and Applied Botany Division, CSIR-Indian Institute of Integrative Medicine, Canal Road, Jammu, 180001 India; 3 Department of Biological Sciences, University of Lethbridge, Lethbridge, AB T1K 3M4 Canada

**Keywords:** Plant stress responses, Plant sciences, Ecology

## Abstract

Bryophytes are potent metal absorbers, thriving well on heavy metal (HM)-polluted soils. Mechanisms controlling uptake, compartmentalization and impacts of HMs on bryophytes life cycle are largely unknown. The current study is an effort to decipher mechanisms of nickel (Ni) excess-induced effects on the phenological events of two bryophytes, *Asterella wallichiana* and *Plagiochasma apendiculatum* growing in natural habitats. Observations revealed Ni-excess induced negative impacts on abundance, frequency of occurrence of reproductive organs, population viability and morphological traits, spore viability and physiological attributes of both the liverworts. Results led us conclude that *P*. *appendiculatum* survived better with the lowest impact on its life cycle events than *A*. *wallichiana* under Ni excess in natural habitats. Our findings collectively provide insights into the previously unknown mechanisms of Ni-induced responses in liverworts with respect to phenological attributes, as well as demonstrate the potential of *P*. *appendiculatum* to survive better in Ni excess habitats.

## Introduction

Uncontrolled urbanization and human interference have tremendously contributed toward releasing toxic elements into the surrounding ecosystems, generating genotoxic stress that causes species extinction and depletion of biodiversity^1–4^. Nickel (Ni) is a non-biodegradable heavy metal (HM), which poses environmental pollution threats, damaging biosphere and human health worldwide^5^. Ni is found in natural soils at trace concentrations [< 0.005–100 parts per million (ppm)], and finds its way into environment through anthropogenic activities like metal smelting, municipal sludge, industrial effluents, fertilizers and pesticides^6^. Ni toxicity is manifested at numerous levels, leading to inhibition of mitosis^7^, compromised plant growth, altered photosynthesis and plant water status, inhibition of Calvin cycle enzyme activities^8^, repression of nitrogen metabolism^9^ and generation of oxidative stress, as well as blockade of adequate absorption of other essential metals^8^. Most common symptoms of Ni phytotoxicity include chlorosis, necrosis, stunting of shoots and roots, and decrease in leaf size^6^. Although it is phytotoxic, Ni forms an important constituent of urease, the sole enzyme known to maintain Ni in stoichiometric proportion in higher plants^8^.

Translocation of HMs from roots to leaves and reproductive organs, and their cumulative effects on the development of reproductive organs in higher plants have been recently studied^10,11^. Reports have indicated that reduced pollen load, delayed or early dehiscence of anthers and disruptive changes in post-fertilization of eggs in higher plants are associated with HM uptake and translocation^12–14^. However, translocation of HMs and their impacts on reproductive abilities of lower plants like bryophytes are currently unknown. So far, a few scientific researches conducted in lower plants like algae^15,16^ and bryophytes^17–19^ have provided very shallow information.

In general, bryophytes are the simplest and most primitive group of land plants. They are divided into three divisions namely liverworts, hornworts and mosses, which differ from each other in terms of morphological features of gametangia and sporophytes^20^. Bryophyte acts as a sink for various HMs, accumulating higher concentrations of metal cations than those found in the associated substrates. The high metal-accumulating capacity of the bryophytes can be attributed to their higher surface-to-volume ratio and frequent absence of the cuticle^21^. HM-induced physiological responses and impacts on the phenological events of these tiny plants are least understood. Among bryophytes, liverworts represent mostly mesic organisms that grow on humus, exposed surfaces of rocks and deserts, and constitute a significant component of vegetation in the temperate biomes^19^. Lack of a significant cuticle and well-developed conducting system has made bryophytes potent metal absorbers, bioindicators and biomonitoring agents^22^.

*Asterella wallichiana* (Lehm. & Lindenb.) Grolle^23^ and *Plagiochasma apendiculatum* Lehm. & Lindenb.^24,25^ are natives of western Himalaya, and represent a group of commonly distributed dioecious liverwort species of the North-Western Himalayas and Western Ghats of India^26^. A few studies on these liverworts have been conducted, mainly for evaluation of HM-induced changes in antioxidant systems of *A*. *wallichiana*^27^ and *P*. *appendiculatum*^28^. Largely, the impacts of HMs on the life cycle (reproductive abilities) of these tiny plants remain least understood, and have so far been ignored. The present study constitutes an effort to bridge the gap of missing information of HM-induced phenological changes in bryophytes. We explored the morphological and physiological, as well as reproductive responses of liverworts toward tolerance to Ni excess on natural sites. Our findings demonstrate profound effects of Ni excess on the life cycle of these bryophyte species and enable us to discover previously unknown mechanisms of Ni responses in these liverworts in a comparative manner.

## Results

### Contents of macronutrients and micronutrients in soil samples collected from the natural habitats and soil HM pollution indices

Soil samples were collected from ten different sites, which were later grouped into two sampling groups on the basis of their nickel (Ni) concentration, namely the control group CS (10.25 ± 0.79 mg kg^−1^) and the Ni excess group NS (125 ± 5.16 mg kg^−1^). In the CS, the macronutrients together with their determined concentrations were organic carbon (C, 4920 ± 9.3 mg kg^−1^), phosphorus (P, 17.27 ± 2.2 mg kg^−1^), organic matter (9145 ± 89.98 mg kg^−1^) and nitrogen (N, 462.8 ± 9.87 mg kg^−1^) (Table 1), whereas the micronutrients along with their concentrations were copper (Cu, 2.89 ± 0.23 mg kg^−1^), zinc (Zn, 1.78 ± 0.49 mg kg^−1^), iron (Fe, 2.12 ± 0.48 mg kg^−1^) and manganese (Mn, 1.23 ± 0.12 mg kg^−1^) (Table 1). In the NS, only significant differences in Ni and P contents were noted when compared with that in the CS. The soil pH values were 8.3 (mild alkaline) and 7.7 (slight alkaline) in the CS and NS, respectively (Table 1).

**Table 1:** Soil analyses of sites inhabited by *Asterella wallichiana* and *Plagiochasma appendiculatum*.

Serial number	Element/matter	Control site (CS)	Ni-excess site (NS)
**Macronutrients (mg kg**^**-1**^**)**			
1	Organic carbon	4920 ± 9.3a	5150 ± 8.3a
2	Phosphorus (P)	17.27 ± 2.2b	11.78 ± 1.2a
3	Nitrogen (N)	462.8 ± 9.87a	442.9 ± 8.26a
4	Organic matter	9145 ± 89.98a	8858 ± 98.21a
**Micronutrients (mg kg**^**-1**^**)**			
1	Copper (Cu)	2.89 ± 0.23a	3.35 ± 0.38a
2	Zinc (Zn)	1.78 ± 0.49a	1.55 ± 0.56a
3	Iron (Fe)	2.12 ± 0.48a	2.28 ± 0.72a
4	Manganese (Mn)	1.23 ± 0.12a	1.79 ± 0.21a
5	Nickel (Ni)	10.25 ± 0.79a	125 ± 5.16b
6	Soil pH	8.3 ± 0.001	7.7 ± 0.001

Concentrations (mg kg^-1^) of macronutrients (organic carbon, phosphorus, nitrogen and organic matter), micronutrients (copper, zinc, iron and manganese) and trace elements (nickel, Ni) present in soil inhabited by *A. wallichiana* and *P. appendiculatum* under field conditions. Samples from five collection sites were individually taken. Sites with lower Ni concentrations (10.25 ± 0.79 mg kg^-1^) were referred as ‘control site’ (CS, *n* = 5) and sites with higher Ni concentrations (125 ± 5.16 mg kg^-1^) were referred as ‘Ni-excess site’ (NS, *n* = 5). Data presented are means ± standard errors. Different letters (a, b) within a row indicate significant differences from each other (Tukey’s test, *P* ≤ 0.05).

The HM contamination in the soils inhabited by the two liverworts was also examined using the standard procedures of soil pollution indices (Table 2). Among the tested soil pollution indices, Ni single pollution index (PI) was found to be the highest for the NS (4.31) compared with that of CS (0.353). The degree of Ni excess of the soil was also evaluated by the contamination factor (Cf) that displayed much higher value in the NS (25) than in the CS (2.05). For Cu and Zn, the PI and Cf values did not show much difference between the CS and NS (Table 2). These data indicated a strong Ni-pollution of the soils in the NS versus CS. This result was further supported by the data of sum of contamination (PI_sum_) and Nemerow pollution index (PI_Nemerow_). PI_sum_ and PI_Nemerow_ showed approximately 10- and 3-times higher scores, respectively, in NS than CS. According to the soil classification based on PI_Nemerow_ index to assess overall degree of soil pollution, the examined NS were placed in the class IV showing moderate level of soil pollution, whereas the CS were placed in the class II with warning limit of soil pollution (Table 2).

**Table 2:** Single pollution index (PI), contamination factor (Cf), sum of contamination (PIsum) and Nemerow pollution index (PI_Nemerow_) of nickel-excess (NS) and control sites (CS).

	Single pollution index (PI)			Contamination factor (Cf)			Sum of contamination (PIsum)	Nemerow pollution index (PI_Nemerow_)
	Ni	Cu	Zn	Ni	Cu	Zn		
Control site (CS)	0.353a	0.07429a	0.01017a	2.05a	0.0578a	0.01017a	0.45269a	0.859a
Nickel-excess site (NS)	4.31b	0.08611a	0.008857b	25b	0.067b	0.008857b	4.41825b	2.412b

For the PI and Cf, concentrations of Ni, Cu and Zn measured in the CS and NS (*n* = 5) were compared with their respective world mean value^37^. PIsum was calculated using the geometric mean of PI of each metal in the CS and NS. Different letters (a and b) within a column indicate significant differences from each other in all combinations (Tukey’s test, P ≤ 0.05).

### Ni uptake potential of liverworts

*Asterella wallichiana* and *Plagiochasma appendiculatum* grown in their natural habitats were examined for their Ni uptake potential at the gametophytic stage. Results showed that Ni uptake potential of *A*. *wallichiana* and *P*. *appendiculatum* varied significantly in NS compared with CS. About 12.6- and 7.7-fold increases in Ni uptake potential (Niup) were noted in the gametophytic thalli of *A*. *wallichiana* and *P*. *appendiculatum*, respectively, in the NS relative to that of the CS under natural conditions (Table 3). These observations provided us a stimulus to investigate the morphological and physiological changes in the gametophytic thalli of *A. wallichiana* and *P*. *apendiculatum* during their life cycle events in response to Ni excess under field conditions.

**Table 3:** Physiological indices of young gametophytic thalli of *Asterella wallichiana* and *Plagiochasma appendiculatum*.

Serial number	Parameter	Control site (CS)	Ni- excess site (NS)
1A	DB	155 ± 6.78a	117.8 ± 4.87b
1P	DB	165 ± 4.54a	148.5 ± 5.67b
2A	Niup	1.25 ± 0.05a	15.8 ± 1.21b
2P	Niup	1.35 ± 0.06a	10.5 ± 0.98b
3A	Ti	1 ± 0.01a	0.79 ± 0.02b
3P	Ti	1 ± 0.01a	0.9 ± 0.01
4A	RSA	135 ± 5.25a	103 ± 3.39a
4P	RSA	185 ± 7.25a	172 ± 4.88a
5A	MDA	0.36 ± 0.04a	0.83 ± 0.03b
5P	MDA	0.21 ± 0.012a	0.42 ± 0.02a
6A	H_2_O_2_	1.13 ± 0.077a	1.32 ± 0.076a
6P	H_2_O_2_	1.09 ± 0.087a	1.22 ± 0.092a
7A	REC %	9.54 ± 1.32a	13.34 ± 1.24b
7P	REC%	12.56 ± 1.19a	13.25 ± 1.21a
8A	Chl *a*	0.685 ± 0.01a	0.558 ± 0.011b
8P	Chl *a*	0.174 ± 0.012a	0.155 ± 0.016a
9A	Chl *b*	0.771 ± 0.03a	0.528 ± 0.022b
9P	Chl *b*	0.625 ± 0.01a	0.498 ± 0.019b
10A	CAR	0.053 ± 0.001a	0.036 ± 0.009b
10P	CAR	0.09 ± 0.002a	0.125 ± 0.003b
11A	ASA	4.9 ± 0.31a	6.9 ± 0.45b
11P	ASA	4.23 ± 0.21a	4.75 ± 0.34a
12A	GSH	2.1 ± 0.098a	2.98 ± 0.087a
12P	GSH	2.48 ± 0.12a	4.3 ± 0.41b
13A	PL	7.8 ± 0.33a	11.4 ± 0.89b
13P	PL	9.8 ± 0.88a	15.4 ± 1.02b

DB (mg kg^-1^, dry weight), dry biomass ; Niup, nickel (Ni) uptake (mg kg^-1^, dry weight); Ti, Ni tolerance index (Ti); RSA, rhizoid surface area; malondialdehye (MDA) and H_2_O_2_ contents (µmol g^-1^ fresh weight); relative electrical conductance (REC %), chlorophyll *a* (Chl *a*), chlorophyll *b* (Chl *b*), carotenoid (CAR), ascorbic acid (ASA), glutathione (GSH) and proline (PL) contents (mg g^-1^ fresh weight) in young gametophytic thalli of *A. wallichiana* and *P. appendiculatum* grown in Ni-excess sites (NS, *n* = 5) and control sites (CS, *n* = 5) were measured. Different letters (a and b) within a row indicate significant differences from each other in all combinations (Tukey’s test, *P* ≤ 0.05). Bold letters A and P represent *A. wallichiana* and *P. appendiculatum*, respectively.

### Effect of Ni excess on the phenological events of liverworts

Both *A*. *wallichiana* and *P*. *appendiculatum* were collected from CS and NS sites in their young gametophytic stages lacked mature antheridia and archegonia. These young gametophytic samples of male and female thalli of *A*. *wallichiana* and *P*. *appendiculatum* were examined for the development of mature antheridia and archegonia, and post-fertilization changes to develop sporophyte, with four visits spreading over the one year period.

#### Effect of Ni excess on abundances of *liverworts*

**Figure 1:**
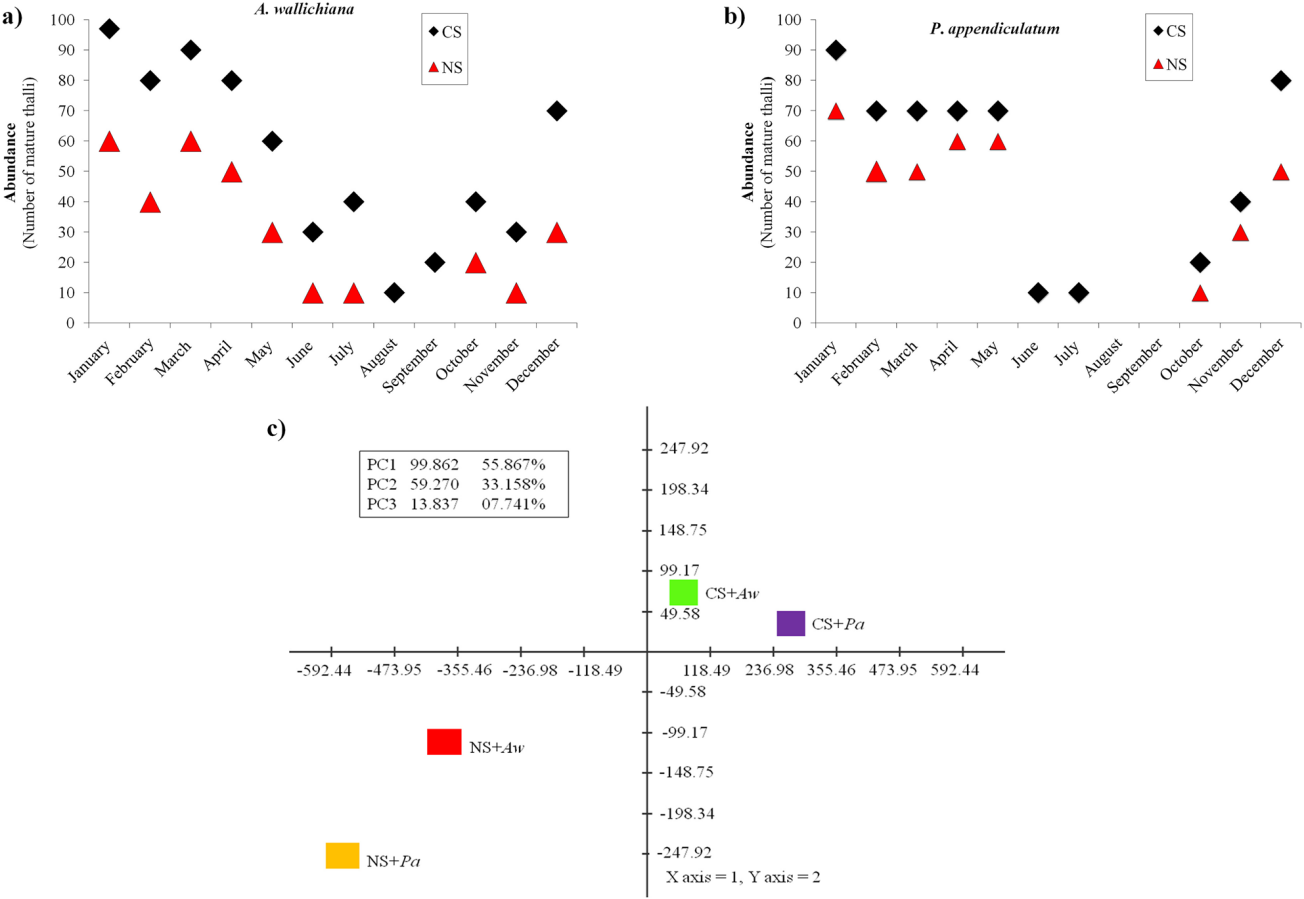
Nickel excess affects abundance of liverworts. **(a,b)** showing impacts of nickel (Ni) excess on the abundance of **(a)** *Asterella wallichiana* and **(b)** *Plagiochasma appendiculatum* in Ni-excess sites (NS, *n* = 5) compared with control sites (CS, *n* = 5) during January 2017 to December, 2017 in natural habitats under field conditions in quadrates (*n* = 10; 10 × 10 cm^2^/quadrat) laid on each of NS and CS, **(c)** principal component analysis showed impacts of Ni excess on variation in abundance of *A*. *wallichiana* and *P*. *appendiculatum* in natural habitats under field conditions in quadrates (*n* = 10; 10 × 10 cm^2^/quadrat) laid on each of NS (*n* = 5) and CS (*n* = 5)*.* CS + *Aw* and CS + *Pa* denote control sites + *A*. *wallichiana* and *P*. *appendiculatum*, whereas NS + *Aw* and NS + *Pa* denote Ni excess sites + *A*. *wallichiana* and *P*. *appendiculatum,* respectively.

The abundances of *A*. *wallichiana* and *P*. *appendiculatum* were counted in terms of the number of mature gametophytic thalli in both NS and CS sites. The impact of Ni excess on the abundances of *A*. *wallichiana* and *P*. *appendiculatum* was examined during a one-year period (January 2017-December 2017) (Fig. 1a,b)*.* In general, *A*. *wallichiana* showed more abundance than *P*. *appendiculatum* throughout the year in CS. However, in NS, *A*. *wallichiana* exhibited a higher reduction in abundance as compared to *P*. *appendiculatum*. The maximum reduction in abundance was observed in December, January, and February month in *A*. *wallichiana* by the difference in the average number mature thalli by 40, 40 and 40, respectively than in *P*. *appendiculatum* by 20, 20 and 30, respectively (Fig. 1a,b). Furthermore, principal component analysis (PCA) also indicated that the variation in the abundances of mature gametophytic thalli was more evident in *P*. *appendiculatum* than in *A*. *wallichiana* grown in Ni excess sites in NS (Fig. 1c).

**Figure 2:**
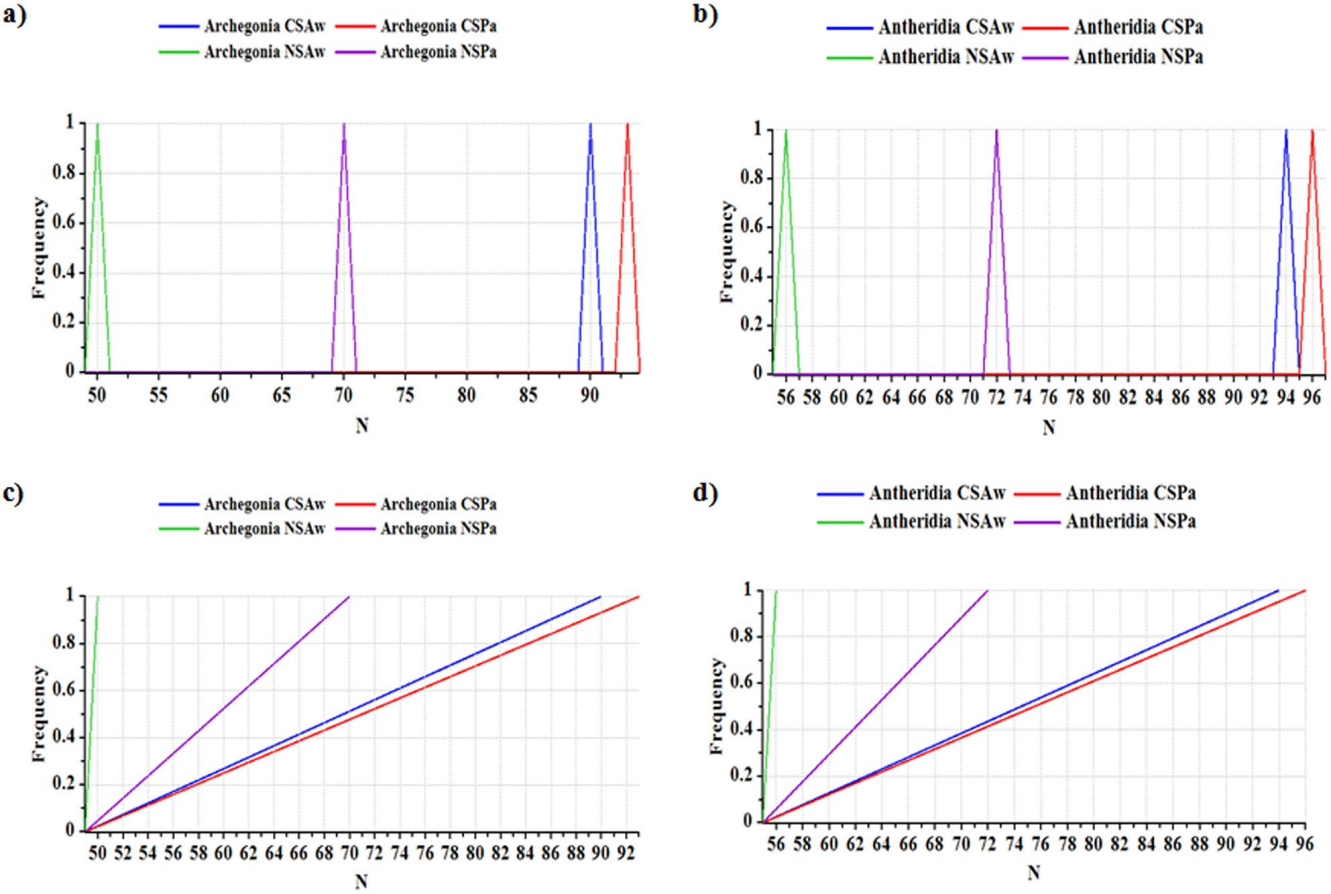
Nickel excess impacts distribution and population viability analysis of liverworts. **(a,b)** Nickel (Ni) in Ni-excess sites (NS, *n* = 5) affected the frequency distribution and number (N) of reproductively active archegonia and antheridia of *Asterella wallichiana* **(a)** and *Plagiochasma appendiculatum* **(b)** compared with the control sites (CS, *n* = 5). X-axis represents number (N) of mature archegonia and antheridia, while Y-axis represents frequency distribution pattern. **(c,d)** Quasi-extinction (QE) probability *versus* threshold curves showed the frequency of occurrence (FOC) of reproductively active antheridia and archegonia as an indicator of population viability of *A*. *wallichiana* and *P*. *appendiculatum*. Green and violet lines show the impact of Ni excess on the FOC of archegonia **(c)** and antheridia **(d)**, respectively in NS compared to blue and red colored lines showing FOC of archegonia **(c)** and antheridia **(d)** in CS. X-axis represents the number (N) of reproductively active antheridia and archegonia, while Y-axis represents the frequency distribution pattern of the *A*. *wallichiana* and *P*. *appendiculatum* in CS and NS sites. ‘Archegonia CS + *Aw*’ and ‘Archegonia CS + *Pa*’ denote ‘control sites + *A*. *wallichiana*’ and ‘control sites + *P*. *appendiculatum,* respectively. ‘Archegonia NS + *Aw*’ and ‘Archegonia NS + *Pa*’ denote ‘Nickel excess sites + *A*. *wallichiana*’ and ‘Nickel excess sites + *P*. *appendiculatum,* respectively. ‘Antheridia CS + *Aw*’ and ‘Antheridia CS + *Pa*’ denote ‘control sites + *A*. *wallichiana*’ and ‘control sites + *P*. *appendiculatum*’, respectively. ‘Antheridia NS + *Aw*’ and ‘Antheridia NS + *Pa*’ denote ‘Nickel excess sites + *A*. *wallichiana*’ and ‘Nickel excess sites + *P*. *appendiculatum*’, respectively.

Population viability analysis (PVA) carried out on the young gametophytic thalli of *A*. *wallichiana* and *P*. *appendiculatum* grown on the CS and NS showed visible differences in the distribution of reproductively active archegonia and antheridia. Transition of the young gametophytic thalli into archegonia and antheridia production was counted during a period of four weeks. During this time, NS showed significant impact on lowering the number (N) of reproductively active archegonia and antheridia in both the liverworts compared to CS (Fig. 2a,b). However, *A*. *wallichiana* exhibited a higher reduction in the number of reproductively active archegonia and antheridia compared to *P*. *appendiculatum* (Fig. 2a,b). Furthermore, the quasi-extinction (QE) (i.e., the probability calculated versus threshold level) also showed a negative effect of Ni excess on the distribution patterns of the reproductively active archegonia and antheridia in both the liverworts (Fig. 2c,d). Importantly, on the basis of the PVA and QE data, effect of Ni excess on the number of reproductively archegonia and antheridia was found to be more evident in *A*. *wallichiana* than in *P*. *appendiculatum* (Fig. 2a–d).

#### Effect of Ni excess on physiological indices

Ni excess showed negative impact on the physiological indices of both the liverworts. Such as, effect of Ni excess was evident on the gametophytic thallus dry biomass (DB), with 24% and 10% reduction in *A*. *wallichiana* and *P*. *appendiculatum*, respectively, recorded in NS compared to CS (Table 3). Ni tolerance index (Ti) in NS was 0.79 (*A*. *wallichiana*) and 0.9 (*P*. *appendiculatum*) as compared with their respective value in CS (Table 3). Rhizoids surface area (RSA) of the *A*. *wallichiana* and *P*. *appendiculatum* gametophytic thalli grown in NS showed reduction by 23.7% and 7%, respectively, compared with their corresponding CS (Table 3). Abiotic stresses exert secondary oxidative damages to plants besides the ionic toxicity, which results in peroxidation of the cell membrane lipids^29^. About 2.3- and 2-fold increase in malondialdehyde (MDA) content was recorded in young gametophytic thalli of *A*. *wallichiana* and *P*. *appendiculatum,* respectively*,* grown in NS compared with CS (Table 3). Additionally, the relative electrolyte conductance (REC), showed maximum increase of 40% and 5% in *A*. *wallichiana* and *P*. *appendiculatum* respectively in NS relative to that of CS (Table 3). H_2_O_2_ content in young gametophytic thalli of both *A*. *wallichiana* and *P*. *appendiculatum* were more in NS compared to CS (Table 3). Among the examined photosynthesis pigments, the levels of chlorophyll (Chl) *a* decreased most significantly by 18% in *A*. *wallichiana* and 11% in *P*. *appendiculatum* in NS when compared with CS (Table 3). A dramatic decrease in Chl *b* content was observed in *A*. *wallichiana* (32%) and *P*. *appendiculatum* (20%) in NS, when compared with CS (Table 3). The carotenoid (CAR) content was decreased in *A*. *wallichiana* by 32% and increased in *P*. *appendiculatum* by 38% in NS compared with CS (Table 3). However, in CS, *P*. *appendiculatum* showed higher CAR content than *A. wallichiana*.

We have also studied the level of non-enzymatic antioxidants such as ascorbic acid (ASA) and glutathione (GSH). About 41% and 12% increase in ascorbic acid (ASA) content was recorded in *A*. *wallichiana* and *P*. *appendiculatum,* respectively, in NS Compared to CS (Table 3). Glutathione (GSH) content was enhanced by 42% and 73% in *A*. *wallichiana* and *P*. *appendiculatum,* respectively*,* in NS as compared to CS. Proline content was increased by 46% and 57% in *A. wallichiana* and *P. appendiculatum* in NS as compared to CS (Table 3).

#### Effect of Ni excess on the development of gametophytic stages of liverworts

A negative correlation was found between Ni uptake and reproductive potential of the gametophytic stages of the two liverworts. Developments of male and female reproductive organs were affected by Ni excess (Table 4). Though no visible changes in the development of antheridia and archegonia were recorded in *A*. *wallichiana* and *P*. *appendiculatum* in both NS and CS, approximately 32% and 21% decrease in frequency of occurrence (FOC) of antheridia (total of antheridia/per 10 × 10 cm^2^ patch) was noted for *A*. *wallichiana* and *P*. *appendiculatum*, respectively (Table 4). While FOC of normal antheridia encounter (NAntE) of *A*. *wallichiana* and *P*. *appendiculatum* declined by 20.7% and 14.4%, respectively, in NS compared to CS. Development of archegonia was also negatively affected in NS (Table 4), about 25% and ~ 13% reduction in FOC of archegonia (total of archegonia/per 10 × 10 cm^2^ patch) was noted for *A*. *wallichiana* and *P*. *appendiculatum*, respectively, relative to that of CS. While, FOC of normal archegonia encounter (NArcE) of *A*. *wallichiana* and *P*. *appendiculatum* showed reduction by 25% and 13%, respectively, in NS compared to CS (Table 4). Spore viability was also reduced in both the liverworts in NS compared to CS (Table 4).

**Table 4:** Nickel impacts frequency of occurrence of antheridia and archegonia and spore viability in *Astrella wallichiana* and *Plagiochasma appendiculatum*.

	Treatment	Antheridia		Archegonia		Spore viability		
		FOC	NAntE (%)	FOC	NArcE (%)	T	V	NV
CS	*A*. *wallichiana*	100	94	100	90	100 ± 6.45	80 ± 5.25	20 ± 1.20
	*P*. *appendiculatum*	100	96	100	93	100 ± 7.25	83 ± 4.15	17 ± 1.76
NS	*A*. *wallichiana*	68	73.32	75	67.5	100 ± 6.38	70 ± 5.98	30 ± 2.88
	*P*. *appendiculatum*	79	81.6	87	80.9	100 ± 5.33	75 ± 5.14	25 ± 2.43

Visible effects of Ni excess (NS, *n* = 5) observed on frequency of occurrence (FOC) of normal antheridia and archegonia of *A. wallichiana* and *P. appendiculatum* compared to control sites (CS, *n* = 5). Data presented are FOC taking control as 100%. *Abbreviations*: FOC, frequency of occurrence; NAntE, normal antheridia encounter; NArcE, normal archegonia encounter; T, total spore count; V, viable spore count; NV, non viable spore count. Spore count values represent 100 spore count made per slide, with three independent biological replicates (*n* = *3*).

### Histochemical localization of Ni ions in mature gametophytic thalli and sporophytes of liverworts

**Figure 3:**
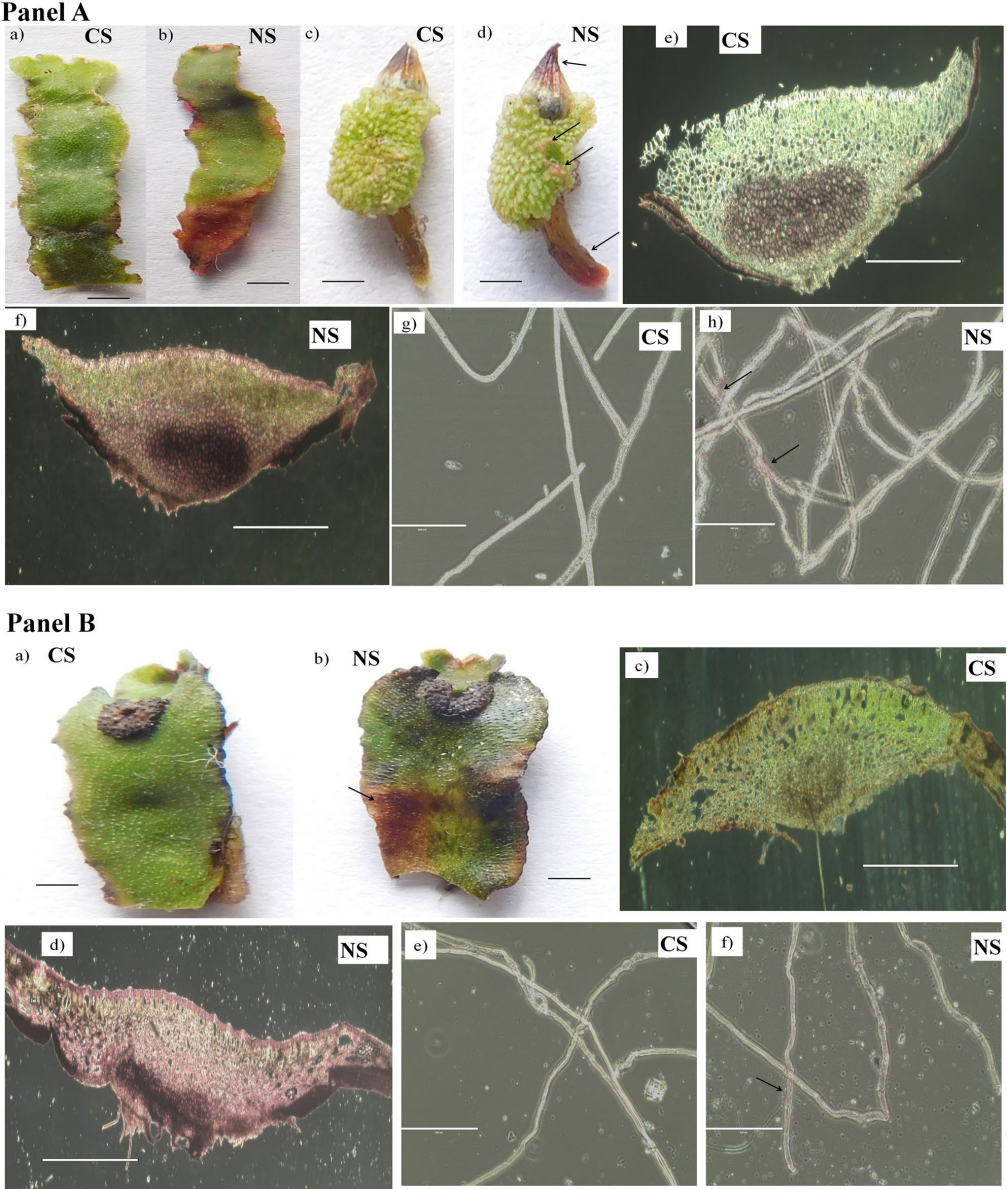
Nickel impacts morphology and reproductive structures of *Asterella wallichiana* and *Plagiochasma appendiculatum*. Panel **(A)**: **(a,b)** *A*. *wallichiana* thalli collected from the Ni-excess sites (NS), **(b)** showed browning of the tissues as an indicator of senescence, compared with that collected from the control sites (CS) **(a)**. **(c,d)** accumulation of Ni ions (dark pink color, arrows) in the *A*. *wallichiana* sporophyte collected from the NS **(d)** compared with those collected from CS **(c)** as revealed by dimethylglyoxime staining. **(e,f)** Transverse section of the *A*. *wallichiana* thalli collected from the NS **(f)** showed higher accumulation of Ni ions (pink color, arrows), compared with that collected from the CS **(e)**. **(g,h)** *A*. *wallichiana* rhizoids collected from the NS **(h)** showed higher accumulation of Ni ions (light pink color, arrows), compared with that collected from CS **(g)**. Panel **(B)**: **(a,b)** *P*. *appendiculatum* thalli collected from the Ni-excess sites (NS) **(b)** showed browning of the tissues as an indicator of senescence, compared with that collected from the control sites (CS) **(a)**. **(c,d)** Transverse section of the *P*. *appendiculatum* thalli collected from the NS **(d)** showed higher accumulation of Ni ions (pink color, arrows), compared with that collected from the CS **(c)**. **(e,f)** *P*. *appendiculatum* rhizoids collected from the NS **(f)** showed higher accumulation of Ni ions (light pink color, arrows), compared with that collected from CS **(e)**. Images of panel **(A)** **(a–d)** and panel **(B)** **(a,b)** were taken by Nikon L840 at 300 dpi. Scale bars represent 1000 µm for panel **(A)** **(e,f)** and panel **(B)** **(c,d)** and 100 µm for panel **(A) (g,h)** and panel **B** **(e,f)**.

The thalli of *A*. *wallichiana* and *P*. *appendiculatum* showed browning of the tissue in NS compared to CS as an indicator of senescence (Fig. 3 Panel A and B (a, b)). Dimethylglyoxime (DMG) staining localized the accumulation of Ni^2+^ ions in the mature gametophytic thalli, mature sporophytes, and rhizoids of *A*. *wallichiana* and *P*. *appendiculatum* grown in the NS and CS. Accumulation of Ni ions in sporophyte of *A*. *wallichiana* in NS was visible in the form of pink color compared to CS (Fig. 3 Panel A (c,d)). Transverse section (T.S.) of thalli of *A*. *wallichiana* and *P*. *appendiculatum* in NS group showed Ni accumulation in the form of pink color compared to CS (Fig. 3 Panel A (e,f) and Panel B (c,d)). Accumulation of Ni was also seen in the rhioizds of both the *A*. *wallichiana* and *P*. *appendiculatum* in NS compared to CS (Fig. 3 Panel A (g,h) and Panel B (e,f)).

### Effect of Ni excess on intracellular localization of ROS in young gametophytic thalli

**Figure 4:**
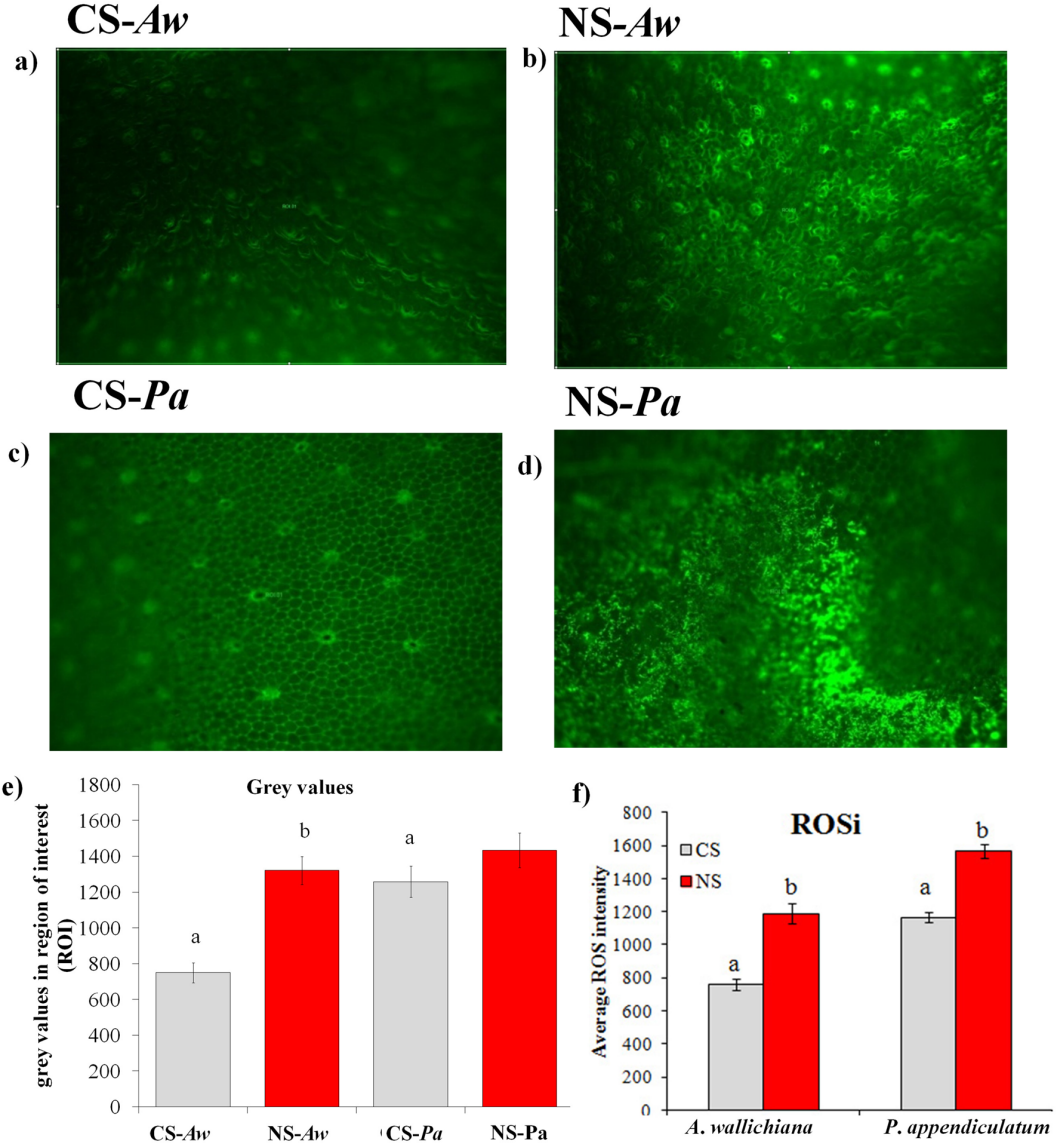
Reactive oxygen species detection in young gametophytic thalli of *Asterella wallichiana* and *Plagiochasma appendiculatum*. 2′,7′-dichlorodihydrofluorescein diacetate (H2DCFDA) based fluorescence microscopy revealed more production of reactive oxygen species (ROS) in the whole thalli of *A*. *wallichiana* and *P*. *appendiculatum* in nickel excess (NS, *n* = *5*) **(b, d)** compared to control sites (**a, c**, respectively) (CS, *n* = *5*); **(e)**, ROS producing regions were imaged and the grey values were calculated by drawing the region of interest (ROI) bearing similar area for the thalli of *A*. *wallichiana* and *P*. *appendiculatum* for Ni excess sites (NS, *n* = 5) and control sites (CS, *n* = 5); **(f)** reactive oxygen species (ROS)-intensity (ROSi) quantified as a function of grey values in young gametophytic thalli of *A. wallichiana* and *P. appendiculatum* grown in control (CS, *n* = 5) and Ni-excess sites (NS, *n* = 5). Data presented are means ± standard errors (*n* = 5). Different letters (a and b) indicate significant differences from each other in all combinations (Tukey’s test, *P* ≤ 0.05).

Results of current study showed higher accumulation of reactive oxygen species (ROS) in both *A*. *wallichiana* and *P*. *appendiculatum* in NS compared to their respective CS (Fig. 4a–d). Grey values measured for the region of interest (ROI) in *A*. *wallichiana* showed 76% increase, while mere 14% increase in grey value was noted in *P*. *appendiculatum* thalli in NS compared to their respective CS (Fig. 4e). On the basis of grey values, reactive oxygen species intensity (ROSi) calculated showed 50% and 33% increase in *A*. *wallichiana* and *P*. *appendiculatum*, respectively, in NS compared to their respective CS (Fig. 4f).

### Ni excess changed urease activity in young gametophytic thalli of *A. wallichiana* and *P. appendiculatum*

**Figure 5:**
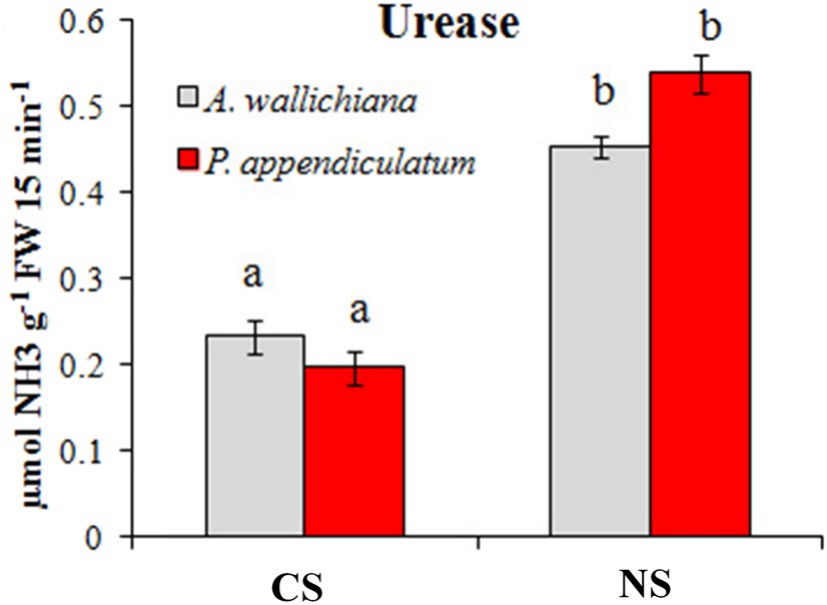
Nickel modulates urease enzyme activity. **(a)** Urease enzyme activity [µmol NH_3_ g^-1^ fresh weight (FW tissue) 15 min^-1^] in thalli of *A. wallichiana* and *P. appendiculatum* grown in control site (CS, *n* = 5) and Ni-excess site (NS, *n* = 5). Data presented are means ± standard errors (*n* = 5). Different letters (a & b) indicate significant differences from each other in all combinations (Tukey’s test, *P* ≤ 0.05).

Urease (EC 3.5.1.5) is a key enzyme regulating the hydrolysis of urea into ammonia and bicarbonate. Understanding the role of urease in facilitating Ni uptake and assimilation into urease could be predicted via determining its activity^30–32^. Results of current study revealed differential responses in terms of urease activity in young gametophytic thalli of *A*. *wallichiana* and *P*. *appendiculatum* grown in NS and CS (Fig. 5). The urease enzyme activity was increased in both the liverworts in NS compared to CS. About 275% increase in urease activity was noted in *P*. *appendiculatum* compared to 96% in *A*. *wallichiana* in NS compared to their respective CS (Fig. 5).

## Discussion

Nickel (Ni) phytotoxicity has been proven to be more disastrous than its deficiency in plants^6^. Soils excess with heavy metals (HMs) like Ni often result in poor plant growth and development^7,8,29–31,33,34^. Plants grown in Ni-excess soils often show reductions in species richness, abundance and plant growth, and increase in membrane damage, and perturbation in physiological mechanisms like photosynthesis and reproductive potential. Current study showed similar impacts of Ni excess on growth, abundance and other physiological and reproductive attributes of both *Asterella wallichiana* and *Plagiochasma appendiculatum* growing in natural habitats.

The HM-uptake potential of a plant depends on several morphological and physiological attributes, such as surface area exposed to HMs, physiological status and thickness of the epidermis^10^. Present study, in natural habitats (Control sites, CS and Nickel excess sites, NS) has shown that *A*. *wallichiana* is endowed with higher Ni-uptake potential than *P*. *appendiculatum*. In general, soil pH has been associated with mobility of metal ions in soil solution and in the rhizosphere zone of a plant^18^. Bryophytes, being hyper-accumulator of metal ions like Cu, Zn, Ni and Pb, uses several mechanisms to reduce metal uptake and/or adsorb metal ions on the surface alone^18^. In the current study, the higher accumulation and availability of more Ni^2+^ ions in NS, *A*. *wallichiana* and *P*. *appendiculatum* could be linked to decrease in pH value from 8.3 in CS to 7.7 in Ni-excess NS (Table 1).

Abundance of higher plants is negatively affected by HMs^35^. Among the lower plants, impact of Ni-Cu complex on boreal forest vegetation has been evaluated along the Russian-Norwegian-Finnish border^36^. Air pollution loaded with Ni has been shown to negatively affect the species richness and abundance of bryophytes. In addition, high pH and high total phosphorous (P) concentrations and low C/N values in the humus have also been advocated as factors causing decline in abundance and species richness of bryophytes^36^. In current study, NS (*n* = *5*) showed reduced abundance of *A*. *wallichiana* and *P*. *appendiculatum* compared with CS (*n* = *5*) (Fig. 1a–c). This finding could be supported by an explanation that the NS having higher Ni excess showed positive correlation with total Cu content and pH value, thus leading to reduced abundance of *A*. *wallichiana* and *P*. *appendiculatum* compared to CS (Table 1).

Soil pollution indices, including PI, Cf, PIsum and PI_Nemerow_, are widely used to evaluate the threat level of a particular element in soil^37^. These indices revealed strong contamination of Ni metal in the NS soils compared with CS soils among the three heavy metals (Cu, Zn and Ni), as only Ni scored higher values of PI, Cf and PI_Nemerow_ in the NS soils than in CS soils (Table 2). Contribution of Ni towards increasing the PIsum value was much higher in the NS than in CS, further supporting that Ni is the only metal causing contamination of the NS soil (Table 2). Cf indices have been used to classify the soils into different categories based on the levels of excess HMs (Cd, Pb, Co, Cr, Ni, V, Cu, Zn, Mo, As, Th, and U)^38^. Application of Cf has also used for the background determination of pollution assessment of HMs in sediments and soils^39^. Similarly, PI_Nemerow_ is used for the assessment of HM contamination in surface layers of Roztocze National Park forest soils (South East Poland)^40^. Additionally, our population viability analysis (PVA) of the data obtained under field conditions also indicated negative impact of Ni excess on the number of reproductively active archegonia and antheridia of both the liverworts, with higher negative Ni effect being observed on *A*. *wallichiana* than *P*. *appendiculatum* (Fig. 2a,b). In support of the PVA result, the Quasi-extinction (QE) analysis also showed negative effect of Ni excess on the numbers of reproductively active archegonia and antheridia of the two liverworts under field conditions, of which *A*. *wallichiana* suffered higher Ni effect than *P*. *appendiculatum* (Fig. 2c,d).

The HM pollution has been shown to induce changes in the surface properties of mosses. A study conducted in *Pleurozium schreberi* (a moss) proved that exposure of moss to Ni could reduce the canopy size^41^. Reductions in rhizoids surface area (RSA) of *A*. *wallichiana* and *P*. *appendiculatum* thalli in NS could be a morphological adaptation of these liverworts to reduce the areas exposed to Ni excess (Table 3). Besides affecting RSA, Ni excess was found to reduce DB and FOC of antheridia and archegonia of both the liverworts, with *A*. *wallichiana* being most affected in the later (Tables 3,4). Successful survival of HM-hyper-accumulator plants grown in HM-excess soils has been linked to their higher tolerance index Ti^42^. For example, higher Ti potential of *Raphanus sativus* L. compared with *Brassica napus* L. grown on multimetal-excess soils advocates its uses in phyto-remediation and better survival in HM excess soils^42^. Higher rhizoids surface area (RSA) has been shown to improve Ti potential by adsorption of metal ions on the surface of rhizoids^18^. Such that, reduced values of Ti for *A*. *wallichiana* in NS could be attributed to more reduction in RSA in comparison with *P*. *appendiculatum* compared to CS (Table 3). The FOC of antheridia and archegonia in both *A*. *wallichiana* and *P*. *appendiculatum* were lower in NS (Table 4), which might be linked to the negative impact of Ni excess on the growth and development of male and female gametopyhtes; and this was more clearly observed in *A*. *wallichiana* than *P*. *appendiculatum*.

Accumulation of HMs and their localization to floral organs have serious implications on reproductive potential of a plant^12^. In *Cucurbita pepo*, HMs (e.g., Zn, Cu, Ni and Pb) were shown to translocate from soil into floral organs, such as pistil, anther and nectary^12^. This HM translocation was found to negatively impact pollen viability, pollen removal and deposition, thereby affecting the overall plant fitness in *C. pepo*^12^. Similarly, HM translocation and accumulation have been shown to impact pollen germination and pollen tube length in tobacco plants^43^. Parallel to these observations, current study also pointed negative impact of Ni excess on the reproductive behavior of *A*. *wallichiana* and *P*. *appendiculatum.* Accumulation of Ni ions in the sporophytes and gametophytes of both the liverworts were observed (Fig. 3 Panel A and B). Prominent changes observed included reduction in the number of FOC of normal antheridia and archegonia in NS, particularly in *A*. *wallichiana* compared to CS (Table 4).

HMs induce production of ROS, which causes extensive damage to lipids, reducing membrane fluidity, and elevates membrane leakiness, as evidenced by increased MDA contents in stressed plants^44^. In current study, a significant increase in MDA content was recorded in young gametophytic thalli of *A*. *wallichiana* and *P. appendiculatum* in NS compared to CS (Table 3). Our findings are in agreement with those of Choudhury and Panda^45,46^, which also observed a gradual increase in MDA content in *Taxithelium nepalense*, a moss subjected to Pb or Cr stress. Besides MDA content, both the liverworts in NS also showed increased membrane leakiness as revealed by higher values of relative electric conductance (REC) in *A*. *wallichiana* than in *P. appendiculatum* compared to CS (Table 3). Observations of fluorescence microscopy revealed that Ni excess in NS could significantly induce ROS production, as evidenced by increases in ROS intensity (ROSi) when compared with CS (Fig. 4a–f).

Photosynthetic pigments are one of the main sites of HM-induced injury in plants^47^. HMs have been shown to reduce photosynthetic pigment (Chl *a* and Chl *b*) contents^44,48^. Ni-induced negative impacts on the photosynthetic pigments were more evident in *A*. *wallichiana* as compared to *P*. *appendiculatum* in NS (Table 3). Higher urease enzyme activity in *P*. *appendiculatum* than *A*. *wallichiana* in NS compared to CS also advocates efficient Ni management by the former than the later (Fig. 5).

The non-enzymatic antioxidant molecules such as glutathione (GSH) and ascorbic acid (ASA), play vital role in tolerance to HMs^49,50^. GSH and ASA both are potential ROS scavenger molecules in plants^49^. In the present study, Ni excess enhanced the GSH and ASA content in both *A*. *wallichiana* and *P*. *appendiculatum*. GSH also acts as a precursor of phytochelatins and helps in the chelation of HMs which are then often sequestered in the vacuoles^50^. *P*. *appendiculatum* showed higher increase in GSH content compared to *A*. *wallichiana* which indicates that *P*. *appendiculatum* has better HMs sequestering and detoxification capacities. The relative abundance of proline is important biochemical indicators for abiotic stress tolerance^51^. Both *A. wallichiana* and *P*. *appendiculatum* accumulated higher proline in NS than CS. Proline regulates the accumulation of usable nitrogen, which might contribute to membrane stability and mitigates the disruptive effect of HMs stress.

**Figure 6:**
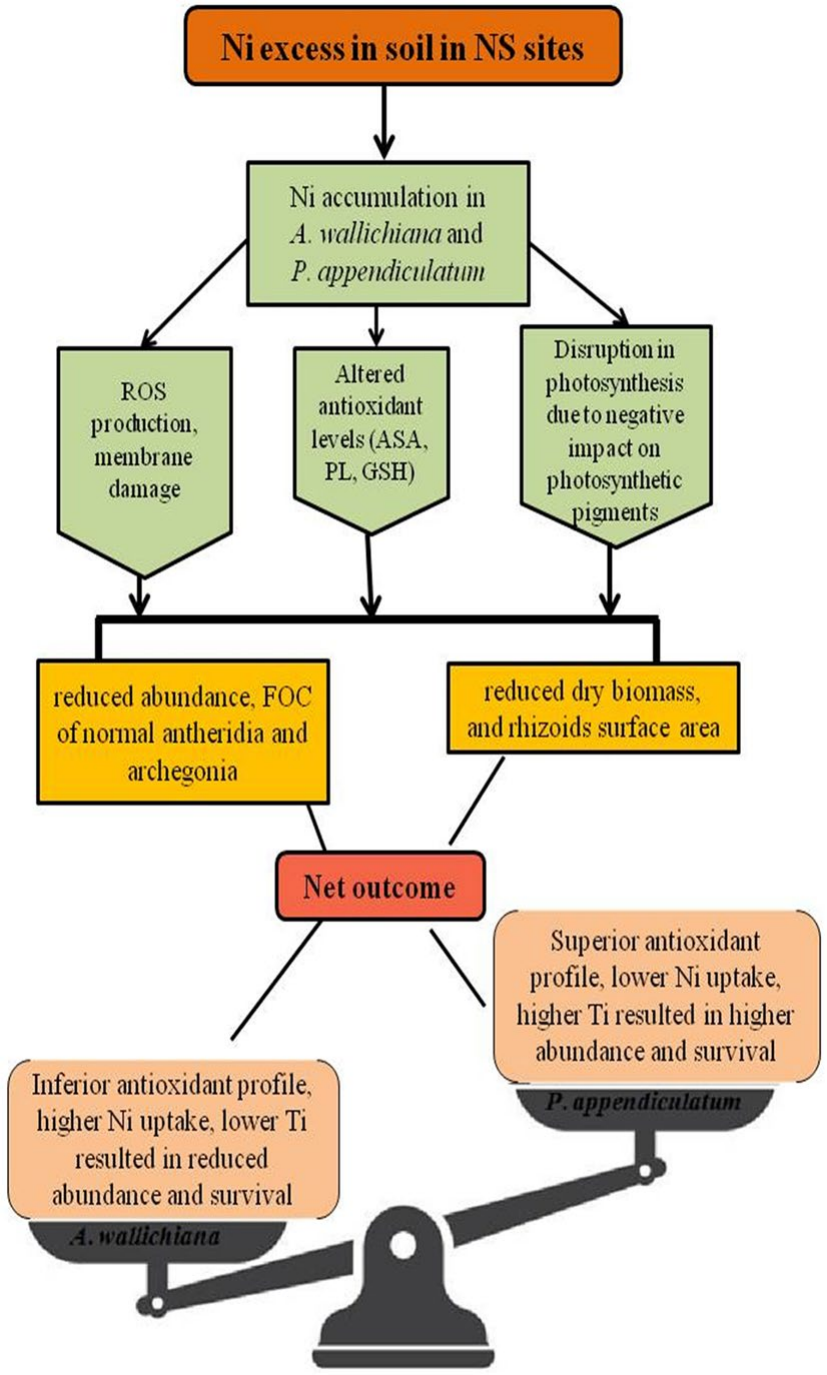
Proposed model representing effects of nickel (Ni) excess in natural habitats on the life cycle events of *Asterella wallichiana* and *Plagiochasma appendiculatum*. Presence of Ni excess in field (NS) conditions resulted in the reduction of overall growth and reproductive performance of both the liverworts. Ni accumulation in thalli of *A*. *wallichiana* and *P*. *appendiculatum* resulted in membrane damage due to excessive production of reactive oxygen species (ROS). Ni excess elevated antioxidant profiles in both the liverworts, especially in *P*. *appendiculatum*. Subsequent effects of Ni accumulation also caused disturbance in nitrogen metabolism (urease enzyme activity), while reduction in photosynthetic pigments in both the liverworts, with higher effects on *A*. *wallichiana* may be linked to reduced photosynthesis. Ni-induced physiological changes led to reduction in dry biomass, rhizoid surface area, abundance, frequency of occurrence of mature gametophytes (male and female) and frequency distribution and number of reproductively active archegonia and antheridia in both two liverworts, with higher effects on *A*. *wallichiana*. Results obtained from comprehensive and comparative field investigations of *A*. *wallichiana* and *P*. *appendiculatum* on NS compared with CS demonstrated that *P*. *appendiculatum* is more capable in terms of Ni excess tolerance compared to *A*. *wallichiana*. *Abbreviations*: reactive oxygen species (ROS), ascorbic acid (ASA), proline (PL), glutathione (GSH), frequency of occurrence (FOC), tolerance index (Ti).

This comprehensive morphological, physiological and reproductive investigations in *A*. *wallichiana* and *P*. *appendiculatum* has helped to understand mechanisms operative in liverworts for the management of Ni-induced oxidative stress under field conditions. Findings are of immense significance in establishing the mechanistic pathway of Ni-induced damage on the life cycles of the liverworts (Fig. 6). The mechanistic model developed on current observations clearly shows that Ni excess can induce morphological, physiological and reproductive changes in *A*. *wallichiana* and *P*. *appendiculatum*. These changes have the potential to negatively impact the phenological events of examined liverworts. Excessive production of ROS under Ni excess in NS, which induced membrane damage, and brought significant changes in *A*. *wallichiana* and *P*. *appendiculatum* and overall plant fitness. Translocations of Ni^2+^ ions and their accumulation in gametophytes impact the maturation of gametophyte to sporophyte.

## Materials and methods

### Experimental sites

The area selected for the present study was district Reasi of the Union territory of Jammu and Kashmir, India which is geographically situated between 33° 4′ 58.1016′′ N latitude and 74° 49′ 59.9268′′ E longitude with an area spanning over 151,701 hectares. Collections of *Asterella wallichiana* (Lehm. & Lindenb.) Grolle and *Plagiochasma apendiculatum* Lehm. & Lindenb. were carried out fortnightly over one year (from January 2017 to December 2017).

### Soil analysis and soil pollution indices of sites inhabited by *A. wallichiana* and *P. appendiculatum*

Soils inhabited by *A. wallichiana* and *P*. *appendiculatum* were collected and analyzed for the presence of various macro- and micronutrients. Estimation of macronutrients in soil samples was performed by the methods of Subbiah and Asija^52^ for nitrogen (N_2_) and Olsen et al.^53^ for phosphorus (P). Determination of micronutrients (Cu, Zn, Fe, Mn and Ni) was estimated by atomic absorption spectroscopy method using standard procedures provided with the instrument (Perkin Elmer 3110, Germany). Soil pollution indices, including single pollution index (PI), contamination factor (Cf), sum of contamination (PIsum) and Nemerow pollution index (PI_Nemerow_), were calculated for Cu, Zn and Ni using the standard procedures described in Qingjie et al.^54^ and Kowalska et al.^37^. These indices were used to classify soils into different classes depending upon the degree of the contamination of a specific HM. For instance, soils with PI < 1, < 2, < 3, < 5 and > 5 indicated absent, low, moderate, strong and very strong soil pollution. For the Cf, soils were classified as follows: Cf < 1 = low contamination, Cf between 1 and 3 = moderate contamination, Cf between 3 and 6 = considerable contamination, Cf > 6 = very high contamination. For PIsum, geometric mean of PI of each HM present in the soil was taken. For PI_Nemerow_, the following criterion was applied for the soil classification: Class I (PI_Nemerow_ values ≤ 0.7), II (PI_Nemerow_ values between 0.7 and 1), III (PI_Nemerow_ values between 1 and 2), IV (PI_Nemerow_ values between 2 and 3) and V (PI_Nemerow_ values > 3). Class I, II, III, IV and V refers to clean, warning limit, slight pollution, moderate pollution and heavy pollution, respectively.

### Nickel uptake potential of *A. wallichiana* and *P. appendiculatum* under field conditions

The young gametophytic stages of both the liverworts collected from the natural sites designated as CS (control sites, S1–S5, thereafter called as CS, *n* = *5*, having minimum Ni concentration) and NS (NS1–NS5, thereafter called as NS, Ni excess sites, *n* = *5*, having high concentration of Ni) sites were washed with tap water followed by distilled water to remove soil particles and other vegetation. To determine the Ni uptake potential of liverworts collected from the field, about 300 mg of oven dried samples of *A*. *wallichiana* and *P*. *appendiculatum* collected from CS and NS were placed in the muffle furnace (300–400 °C for 5 h) to ash, which was then digested using wet-digestion procedure in a mixture of HNO_3_ and HClO_4_ (4:1, v/v) as described elsewhere. The concentrations of Ni (in mg kg^−1^ tissue) were determined using Atomic Absorption Spectrometry (Shimazdu, AA7000, Japan) following manufacturer instructions.

### Collection of young gametophytic thalli of *A. wallichiana* and *P. appendiculatum*

Fresh samples of *A*. *wallichiana* and *P*. *appendiculatum* at young gametophytic stage (marked with absence of mature antheridia and archegonia) were collected from the CS (*n* = 5) an NS (*n* = 5) in district Reasi and brought to the laboratory in polyethylene bags. At this stage, samples were divided into two parts for short-term physiological and biochemical, and long-term morphological analyses:

(i) Short-term analyses: For short-term physiological analyses, gametophytic stages of *A*. *wallichiana* and *P*. *appendiculatum* collected from NS and CS were used for physiological and biochemical parameters such as malondialdehye content (MDA, µmol g^-1^ fresh weight), H_2_O_2_ content (µmol g^-1^ fresh weight) and relative electric conductance (REC%)^29,55^.

(ii) Long-term analyses were carried on the young gametophytic thalli of *A*. *wallichiana* and *P*. *appendiculatum* growing on NS and CS. These sites were visited every week for a period of 1–2 months to observe the development of young gametophyte into mature gametophytes bearing antheridia and archegonia, and development of sporophyte.

### Population viability analysis of *A. wallichiana* and *P. appendiculatum* under field conditions

For measuring the impact of Ni excess on the survival and normal functioning of the reproductive structures (antheridia and archegonia) of *A*. *wallichian*a and *P*. *appendiculatum*, a comprehensive population viability analysis (PVA) was carried using the Vortex ver. 10.3.1^56^. The impact of Ni excess on the frequency distribution and number of the reproductively active archegonia and antheridia of the *A*. *wallichiana* and *P*. *appendiculatum* was determined for the young gametophytic thalli of the two liverworts growing in CS and NS. Vortex was also used to determine the quasi-extinction risk imposed by Ni using several input parameters, such as initial population size (number of mature gametophytes of *A*. *wallichiana* and *P*. *appendiculatum* in CS and NS), mortality (number of gametophytes perished under Ni excess in NS), catastrophe (Ni excess considered as a catastrophe reducing number of reproductively active antheridia and archegonia in NS compared to CS) and reproductive potential of the antheridia and archegonia of both the liverworts.

### Phenological attributes of *A. wallichiana* and *P. appendiculatum* in field conditions

For field conditions, ecological attributes of the habitats of two liverworts, including mean temperature and mean relative humidity (Supplementary Table 1) and pH were recorded. Developmental stages of the liverworts: vegetative (young gametophyte) and reproductive stages (mature gametophyte bearing antheridia and archegonia) and abundance, frequency of occurrence (FOC, number of reproductive structures encountered per 10 × 10 cm^2^ patch of area under study) were recorded. Plants were photographed in the field using a digital camera (Cyber-shot DSC-H10, Sony, USA). Each sample was divided into two parts; one part was kept for the preparation of herbarium and another for the identification of thalli using gametophytic and sporophytic characters.

### Dimethyl glyoxime test for Ni absorption in young and mature gametophytic thalli and sporophytes

To visualize Ni^2+^ ions absorption and its accumulation in *A*. *wallichiana* and *P*. *appendiculatum* in thalli, sporophyte and rhizoids, the dimethyl glyoxime (DMG) staining procedure was used^57,58^. In brief, *A*. *wallichiana* and *P*. *appendiculatum* gametophyte and sporpophytic stages collected from CS and NS were thoroughly washed with double distilled water. Later, tissues were air dried and then placed in petri plates containing dimethyl glyoxime (DMG) solution for 10 min, followed by washing with distilled water to remove any surface retention of DMG. Accumulation of Ni^2+^ ions upon DMG staining was observed as pink color.

### Morphometeric and reproductive parameters in mature gametophytic thalli and sporophytes

The male and female mature gametophyte developmental attributes like archegonia and antheridia numbers were counted and dry biomass was measured. Sporophyte viability was determined by staining the spores with 2,3,5-triphenyltetrazolium chloride (TTC). For anatomical studies, ventral section of thallus stained with DMG for CS and NS of both the *A*. *wallichiana* and *P*. *appendiculatum* were cut manually. Sections were mounted in glycerin before photomicrography using a NIKON ECLIPSE E400 (Nikon Corporation, Tochigi, Japan) camera.

### Stress indicators

#### Reactive oxygen species measurement in young gametophytic thalli

Reactive oxygen species (ROS) detection in young gametophytic thalli of *A*. *wallichiana* and *P*. *appendiculatum* was done using 2′,7′-dichlorodihydrofluorescein diacetate (H_2_DCFDA) based fluorescence microscopy^59^. Briefly, the thalli were placed on glass Petri plate containing Ni solution (0.1 mM, mocking NS habitat, NS) for 10 min and distilled water (mocking control habitat, CS). They were then allowed to float on a 60 µM H_2_DCFDA solution prepared in buffer (1 mM KCl, 1 mM MgCl 2 and 5 mM MES, pH 6.1) for 10 min in dark. After a brief wash with buffer, the thalli were observed using Leica DM1000 fluorescence microscope under GFP filter having 470/40 nm bandpass excitation and emission of 525 nm fluorescence microscopy. The whole thalli were imaged, and the grey values were calculated by drawing ROI (region of interest) bearing similar area for all the samples. The average intensities were used to calculate the ROS concentration.

#### Non-enzymatic antioxidant profiles of *A*. *wallichiana* and *P*. *appendiculatum*

Estimations of ascorbic acid (ASA), glutathione (GSH) and proline (PL) were done as described in Choudhary et al.^29^.

#### Estimation of Ni-specific metalloenzyme urease (EC 3.5.1.5) activity

Among the Ni-specific metalloenzymes, urease enzyme activity was estimated in *A*. *wallichiana* and *P*. *appendiculatum* collected from the NS and CS following the method of Kandeler and Gerber^32^.

### Statistical analysis

Otherwise stated, for each experiment, five biological repetitions were designed, and the resulting data were expressed as mean values ± standard errors. In entire experiments, each biological repetition had three technical repeats. One-way analysis of variance (ANOVA) was carried out, and data were presented at a significance of *P* ≤ 0.05. Principal component analysis (PCA) was carried using meV software version 4.0^60^.

## Supplementary information

Supplementary Information.
