# Supplementary material for: Nickel excess affects phenology and reproductive attributes of Asterella wallichiana and Plagiochasma appendiculatum growing in natural habitats
Source: Sci Rep. 2021 Feb 9;11:3369. doi: 10.1038/s41598-020-73441-1 (PMC7873240; doi:10.1038/s41598-020-73441-1)
Supplement: Supplementary file 1 — Supplementary Information. [file 41598_2020_73441_MOESM1_ESM.doc]

**Supplementary Table 1. Mean relative humidity range (% age) and mean temperature range (° C) of field conditions.**

|  | **Mean RH (%)**  ***A*. *wallichiana*** | **Mean temperature (° C)**  ***A*. *wallichiana*** | **Mean RH (%)**  ***P*. *appendiculatum*** | **Mean temperature (° C)**  ***P*. *appendiculatum*** |
| --- | --- | --- | --- | --- |
| **January** | 68 | 11 | 68 | 11 |
| **February** | 71 | 16.6 | 71 | 16.6 |
| **March** | 58 | 17.2 | 58 | 17.2 |
| **April** | 52 | 25.1 | 52 | 25.1 |
| **May** | 55 | 32.7 | 55 | 32.7 |
| **June** | 68 | 34.5 | 68 | 34.5 |
| **July** | 75 | 33.3 | 75 | 33.3 |
| **August** | 87 | 30.4 | 87 | 30.4 |
| **September** | 70 | 30.6 | 70 | 30.6 |
| **October** | 63 | 26.8 | 63 | 26.8 |
| **November** | 59 | 21.9 | 59 | 21.9 |
| **December** | 57 | 20.4 | 57 | 20.4 |
